# A conserved residue in the histidine kinase BceS tunes bacitracin stress responses in Bacillus subtilis

**DOI:** 10.1099/acmi.0.001145.v3

**Published:** 2026-08-12

**Authors:** Kate Gridley, Alan Koh

**Affiliations:** 1 Department of Life Sciences, University of Bath, Bath, UK; 2 Milner Centre for Evolution, University of Bath, Bath, UK

**Keywords:** antimicrobial peptide, BceRS-BceAB, flux-sensing, histidine kinase, signal transduction, two-component systems

## Abstract

Two-component systems regulate bacterial responses through histidine kinases (HKs), where the majority act as both a kinase and a phosphatase. In *Bacillus subtilis*, the BceRS-BceAB system mediates bacitracin resistance, but it is unknown whether its HK, BceS, also functions as a phosphatase. Here, we identify the conserved motif within the BceS DHp domain and show that Thr-128 contributes to maintaining kinase–phosphatase balance. Substitutions at this site impaired BceS signalling and bacitracin resistance. Our findings demonstrate that in BceS, Thr-128 is important for proper BceAB regulation.

## Data Summary

The authors confirm that all supporting data and protocols have been provided within the article or through supplementary data files.

## Introduction

**Fig. 1.**
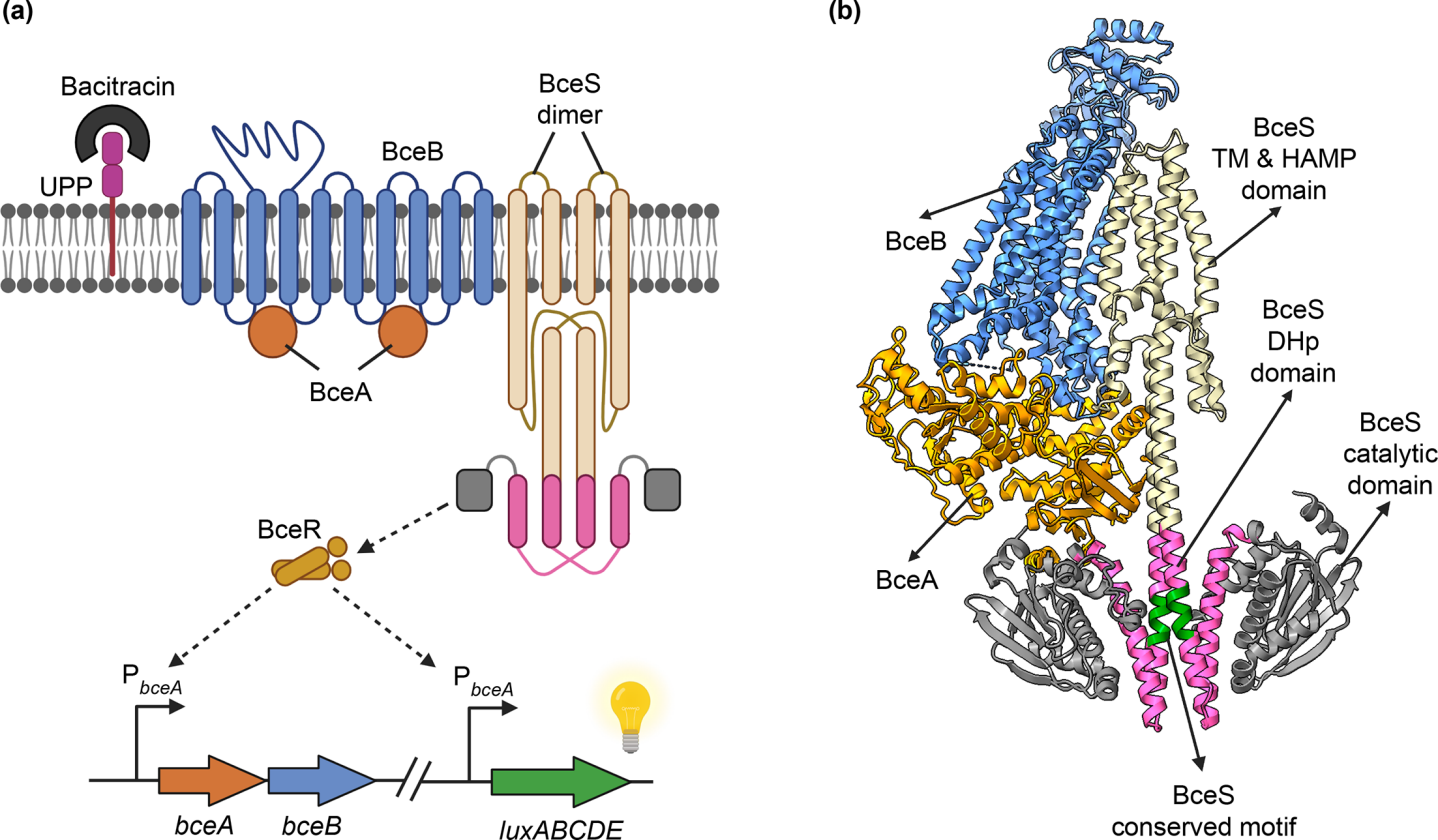
BceRS-BceAB topology. (**a**) Schematic representation of the BceRS-BceAB complex in the presence of extracellular bacitracin. BceB (blue) forms a complex with BceA (orange) and interacts with the HK BceS (beige). Bacitracin (black) binds to its cellular target, undecaprenyl-pyrophosphate (UPP; purple), at the membrane. Upon detection of bacitracin via flux communication, the BceAB transporter activates BceS, which subsequently activates the response regulator BceR (yellow). Activated BceR induces transcription from the *P_bceA_* promoter, leading to the production of additional BceAB transporters. Because BceAB expression is proportional to the bacitracin concentration at the cell membrane, the *P_bceA_*-luciferase reporter can be used to monitor transporter activity. (**b**) BceAB-BceS model (PDB 8G3A), comprising a BceAB transporter containing two BceA subunits (orange) and one BceB subunit (blue), coupled to a BceS dimer. BceS contains the transmembrane and HAMP domain (residues 1–114, beige), the DHp domain (residues 115–172, pink), the catalytic domain (residues 173–334, grey) and the conserved H-E/DxxT/N-PL motif (residues 124–130, green).

Two-component systems (TCSs) are bacterial signalling mechanisms which enable adaptation to environmental stresses. They typically comprise a membrane-bound histidine kinase (HK), which detects external stimuli and a cytoplasmic response regulator that modulates gene expression [1, 2]. HKs contain two conserved domains: a dimerization and histidine phosphotransfer (DHp) domain and a catalytic ATP-binding (CA) domain (Fig. 1a, b) [1, 2]. During signalling, the *γ*-phosphate from ATP bound at the CA domain is transferred to a conserved histidine within the DHp domain, which subsequently transfers the phosphoryl group to the cognate response regulator [1, 2]. Because signalling output depends on the ratio of phosphorylated to unphosphorylated response regulator, this balance must be tightly controlled. To achieve this, some HKs rely on auxiliary phosphatases, while other HKs possess inherent phosphatase activity, allowing direct dephosphorylation of their response regulator to fine-tune signalling [1–3]. In *Bacillus subtilis*, the HK BceS is thought to share this phosphatase function but has not yet been experimentally validated.

BceS functions as part of the BceRS-BceAB module, which confers resistance to the antibiotic bacitracin [4–8]. Unlike canonical TCSs, BceS does not directly sense the stimulus but forms a sensory complex with the ABC transporter BceAB to sense bacitracin stress (Fig. 1a, b) [4, 6, 8–11]. Through a flux-sensing mechanism, BceS activation is proportional to transporter activity and thus reflects extracellular bacitracin levels [9]. Activated BceS phosphorylates the response regulator BceR, which induces expression of the *bceAB* operon (Fig. 1a) [4, 7]. This positive feedback increases BceAB production, enabling efficient detoxification by releasing bacitracin from undecaprenyl-pyrophosphate (UPP) at the membrane surface [5, 9, 12].

As with many HKs, BceS is expected to exhibit dual enzymatic activity, functioning both as a kinase that phosphorylates and as a phosphatase that dephosphorylates BceR. This duality ensures rapid signal tuning, signalling fidelity and minimizing crosstalk between parallel systems [1, 3, 13]. These activities are mediated by conserved residues within the DHp domain, organized around an H-E/DxxT/N-PL motif [3, 14]. Within this motif, acidic residues (glutamic acid, E; aspartic acid, D) and polar residues (threonine, T; asparagine, N) contribute to kinase and phosphatase functions, respectively [3, 14]. While this motif has been well characterized in other HKs, its presence and functional relevance in *B. subtilis* BceS remain to be elucidated. To address this, we investigated whether BceS harbours this conserved motif and evaluated the functional importance of the predicted phosphatase residue within its DHp domain.

## Results

### BceS possesses the conserved H-E/DxxT/N-PL motif

**Fig. 2.**
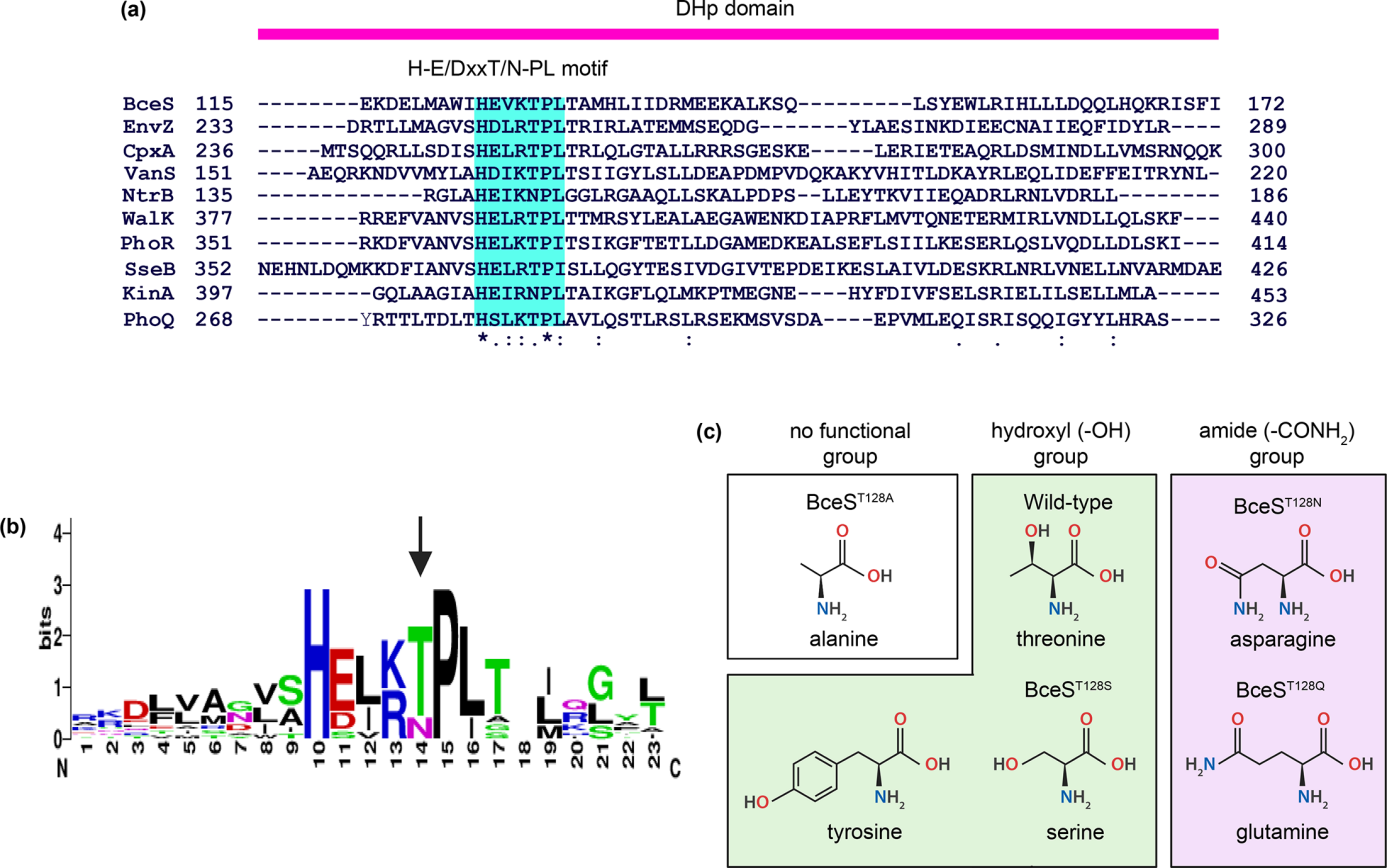
BceS contains the conserved motif in the DHp domain. (**a**) Sequence alignment of the DHp domain of BceS with other HKs was performed with MAFFT [15]. The BceS DHp domain contains the conserved H-E/DxxT/N-PL motif (highlighted in blue). UniProt identifiers of HKs used in this alignment are BceS (O35044), EnvZ (P0AEJ4), CpxA (P0AE82), VanS (Q06240), NtrB (P0AFB5), WalK (Q45614), PhoR (P23837), SseB (Q9L523), KinA (P16497) and PhoQ (P23837). (**b**) Sequence conservation of the phosphatase motif across the analysed HKs. The black arrow indicates the residue implicated for phosphatase activity (BceS, Thr-128). (**c**) Structures of the amino acids chosen for substitution of the BceS residue Thr-128, comprising alanine, serine, asparagine and glutamine.

To determine whether the BceS DHp domain contains the conserved H-E/DxxT/N-PL motif, multiple sequence alignment of the DHp domain from previously characterized HKs was performed with MAFFT (Fig. 2a) [15]. The analysis indicates that BceS harbours the canonical H-E/DxxT/N-PL motif and identified threonine at position 128 as the residue potentially necessary for phosphatase activity (Fig. 2a, b).

### BceS Thr-128 contributes to maintaining kinase–phosphatase balance

To assess whether the Thr-128 residue is critical for BceS phosphatase function, signalling assays were performed in a system where the native *bceS* gene was deleted, and each BceS variant was generated by site-directed mutagenesis and expressed from a single-copy ectopic construct. Activity was monitored *in vivo* using a *P_bceA_-luxABCDE* reporter fusion construct, which measures activation of the *P_bceA_* promoter in response to bacitracin, and by determining MICs.

Substitutions at Thr-128 were anticipated to modulate phosphatase activity, leading to either increased MIC and BceS signalling if dephosphorylation was impaired or decreased MIC and BceS signalling if phosphatase activity was enhanced. Alternatively, a substitution could perturb helix packing within the DHp domain, altering interactions with the CA domain or the response regulator and thereby directly affecting kinase activity. In this scenario, decreased MIC and BceS signalling would likely reflect reduced kinase activity rather than enhanced phosphatase activity. These events are not mutually exclusive, and altered kinase and phosphatase activities could occur simultaneously.

**Fig. 3.**
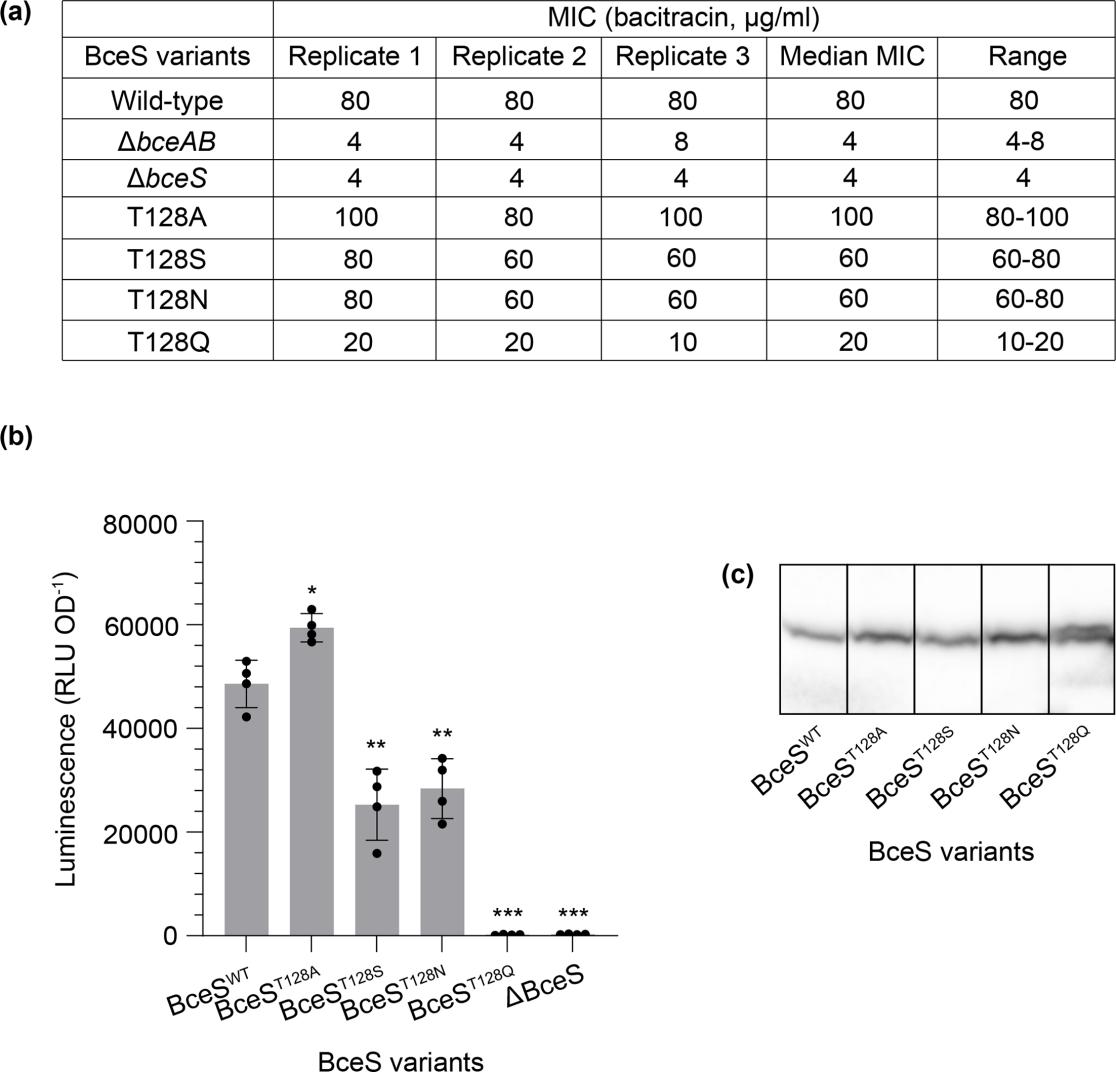
BceS substitution mutants’ signalling activity. (**a**) MIC analysis of BceS substitution mutants carrying the *PbceA-luxABCDE* reporter construct in response to bacitracin. MIC was defined as the lowest bacitracin concentration at which no visible growth was observed. MIC values are from three biological repeats. (**b**) Signalling activity of BceS substitution mutants carrying the *P_bceA_-luxABCDE* reporter construct. Data are shown as mean±sd from four biological repeats. Statistical significance was assessed using Welch’s one-way ANOVA [W (5,7.770)=370.8, *P*<0.0001], followed by Dunnett’s T3 multiple comparisons test (analysed values; Fig. S1, available in the online Supplementary Material). Symbols represent multiplicity-adjusted *P*-values: ***, *P*<0.001; **, *P*<0.01; *, *P*<0.05. (**c**) Western blot analysis of His-tagged BceS substitution mutants detected with *anti-His* antibodies to confirm protein stability (uncropped Western blot; Fig. S2).

The role of the hydroxyl group at position 128 within the BceS DHp domain was examined by considering two other alternative residues containing a hydroxyl functional group, serine and tyrosine. Tyrosine was excluded from the analysis due to its bulky, rigid aromatic ring, which would likely introduce steric hindrance and disrupt DHp domain structure (Fig. 2c). Serine, on the other hand, is smaller and more flexible, potentially reducing steric crashes (Fig. 2c). In the BceS^T128S^ variant, the kinase–phosphatase balance was disrupted, as indicated by reduced MIC and BceS signalling (Fig. 3a, b). This is likely due to an altered orientation of the hydroxyl group and suboptimal DHp domain positioning caused by the smaller side chain.

Within the conserved motif, asparagine, an amide-containing residue, is also frequently conserved in the place of threonine at position 128 (Fig. 2b). To investigate whether hydroxyl and amide groups are functionally interchangeable in BceS, two residues containing amide functional groups, asparagine and glutamine, were investigated. The former is smaller and more compact, while the latter is bulkier and has a longer side chain (Fig. 2c). Similar to the BceS^T128S^ variant, the BceS^T128N^ variant showed a reduced MIC and BceS signalling, suggesting functional compatibility but an altered kinase–phosphatase balance compared to threonine (Fig. 3a, b).

In contrast, substitution with glutamine, giving rise to the BceS^T128Q^ variant, resulted in a dramatic reduction in MIC and abolished BceS signalling to a level that is similar to the *ΔbceS* strain (Fig. 3a, b). The severe phenotype likely arose from the bulky side chain causing steric clashes within the DHp helices, thereby destabilizing the domain (Fig. 2c). Importantly, the loss of signalling in the BceS^T128Q^ variant is not due to detectable protein degradation (Figs. 3c and S2).

To directly assess the importance of the functional groups, alanine was introduced at position 128. Alanine lacks both hydroxyl and amide groups but, due to its small size, will minimally perturb protein structure (Fig. 2c). The BceS^T128A^ variant exhibited a modest increase in MIC and BceS signalling, consistent with an impaired kinase–phosphatase balance (Fig. 3a, b).

## Discussion

In *B. subtilis*, the HK BceS functions as part of the BceRS-BceAB module, which confers resistance to the antibiotic bacitracin [4–8]. Like other HKs, BceS is thought to possess intrinsic phosphatase activity, although this had not yet been experimentally demonstrated. In this study, we demonstrated that BceS harbours the conserved H-E/DxxT/N-PL motif within its DHp domain and that Thr-128 contributes to maintaining proper kinase–phosphatase balance. While hydroxyl and amide groups at Thr-128 may partially substitute for one another, these substitutions can also introduce structural changes that influence kinase function. Consequently, we cannot determine whether the observed reductions in MIC and BceS signalling with serine, asparagine or glutamine substitutions reflect enhanced phosphatase activity, reduced kinase activity or a combination of both.

This uncertainty reflects the limitations of the assays employed in this study. The BceS signalling and MIC assays provide only indirect evidence that Thr-128 is important for BceS function. Although the observed substitution mutant phenotypes are consistent with disruption of the kinase–phosphatase balance, they do not directly demonstrate BceS-mediated dephosphorylation. Direct biochemical assessment of BceS phosphatase activity will therefore need to be tested with a phosphorylation decay assay. In addition, introducing the Thr-128 substitutions into a kinase-deficient BceS variant that retains phosphatase activity could help distinguish whether the observed phenotypes arise from altered kinase activity, phosphatase activity or the balance between the two.

Nevertheless, threonine appears to satisfy both the structural and functional requirements at this position, whereas substitution with other residues disrupts the kinase–phosphatase balance to varying degrees and consequently impacts bacitracin resistance.

Taken together, this work provides a framework for future investigation into the kinase and phosphatase functions of BceS and its interactions with related Bce-like modules, such as PsdRS-PsdAB and YxdJK-YxdLM, where crosstalk between these systems has been observed [4]. Understanding how BceS coordinates with these modules will be critical for defining the broader regulatory networks underlying antimicrobial resistance in *B. subtilis*.

## Experimental procedures

### Strains and growth conditions

All strains used in this study are listed in Table S1 and were routinely maintained and grown at 37 °C in lysogeny broth (LB; 5 g l^−1^ yeast extract, 5 g l^−1^ NaCl, 10 g l^−1^ tryptone) and on LB agar containing 15 g l^−1^ agar. Selective media contained chloramphenicol (5 µg ml^−1^), macrolide-lincosamide-streptogramin B (lincomycin, 25 µg ml^−1^; erythromycin, 1 µg ml^−1^), spectinomycin (100 µg ml^−1^) or ampicillin (100 µg ml^−1^).

### Strain constructions and molecular cloning

All plasmids and primers used in strain construction are listed in Tables S2 and S3, respectively. Site-directed mutagenesis was performed using the QuikChange II Site-Directed Mutagenesis Kit (Agilent Technologies) with plasmid pSD2E01 as the template. All constructs were verified by DNA sequencing. *B. subtilis* transformations with linearized plasmid DNA were carried out as previously described [16].

### Bioinformatic analysis

Sequence alignment of the DHp domain of known and putative HKs was analysed using multiple sequence alignment to identify conserved motifs with MAFFT-DASH [15]. The UniProt identifiers for the analysed proteins are BceS; O35044, EnvZ; P0AEJ4, CpxA; P0AE82, VanS; Q06240, NtrB; P0AFB5, WalK; Q45614, PhoR; P23837, SseB; Q9L523, KinA; P16497 and PhoQ; P23837.

### MIC assay

The sensitivity of *B. subtilis* strains to bacitracin was assessed by growing strains overnight in Mueller–Hinton medium at 37 °C. The following day, cultures were diluted 1 : 500 into 2 ml of fresh medium containing Zn²^+^-bacitracin (0, 2, 4, 8, 10, 20, 40, 60, 80, 100 and 200 µg ml^−1^). Cultures were incubated at 37 °C with shaking at 180 r.p.m. for 24 h. Growth was evaluated, where the MIC was defined as the lowest concentration of bacitracin at which no visible growth was observed. Three independent biological replicates were performed.

### BceS signalling assay

Luciferase activity was measured using a Tecan Spark microplate reader controlled by SparkControl^™^ V2.1 software. Overnight starter cultures were grown at 37 °C in 2 ml LB medium, then diluted 1 : 1000 into fresh medium the following day. One hundred microlitres of diluted cultures were transferred into black, clear-bottom 96-well plates (Corning). Cultures were incubated at 37 °C with continuous orbital shaking at 180 r.p.m. until early exponential phase (OD₆₀₀; 0.3–0.4), at which point Zn²^+^-bacitracin was added to the medium to achieve a final concentration of 1 µg ml^−1^. Optical density (OD₆₀₀) and luminescence (integration time 1 s) were recorded every 5 min. Background correction was performed using LB-only wells, and relative luminescence units (RLU) were normalized to cell density (RLU/OD₆₀₀). Reported values correspond to steady-state activities reached 60–75 min after bacitracin addition. Four independent biological replicates were performed.

### Western blot analysis

Protein levels were measured by growing strains in accordance with the BceS signalling assay. Proteins were harvested as previously described [11] and separated by electrophoresis at 120 V for 50 min with a RunBlue^™^ TEO-Tricine SDS mini gel in Teo-Tricine-SDS (Expedeon) buffer. Separated proteins were transferred to PVDF membranes [Merck Millipore, activated in 100% (v/v) methanol] using a PerfectBlue^™^ Sedec^™^ Semi-Dry Electroblotter (Peqlab) in 1× RunBlue SDS Running Buffer (Expedeon) with 20% (v/v) methanol for 70 min at 75 mA. PVDF membranes containing transferred proteins were blocked for 2 h at room temperature with 7.5% (w/v) skimmed milk powder in PBST [137 mM NaCl, 10 mM Na_2_HPO_4_, 2.7 mM KCl, 1.8 mM KH_2_PO_4_ (pH 7.4), 0.1% (v/v) Tween 20]. The PVDF membranes were then washed three times in PBST and incubated with the primary antibody (Penta·His Antibody, QIAGEN, 1 : 2000 dilution) in PBST (1% bovine serum albumin) overnight at 4 °C. The following day, membranes were washed three times in PBST before incubation with the secondary antibody (Peroxidase-AffiniPure Goat Anti-Rabbit IgG, Jackson ImmunoResearch Europe Ltd, 1 : 4000 dilution) in milk-PBST for 1 h at room temperature, followed by three final washes in PBST. Proteins were detected by chemiluminescence with Pierce^™^ ECL Western Blotting Substrate (Thermo Fisher Scientific Inc.) and a Qiagen CCD camera with Fusion software.

## Supplementary material

10.1099/acmi.0.001145.v3 Supplementary Material 1.

## Abbreviations

CA: catalytic ATP-binding
DHp: dimerization and histidine phosphotransfer
HK: histidine kinase
LB: lysogeny broth
RLU: relative luminescence unit
TCSs: two-component systems
UPP: undecaprenyl-pyrophosphate
