# Supplementary material for: A conserved residue in the histidine kinase BceS tunes bacitracin stress responses in Bacillus subtilis
Source: Access Microbiol. 2026 Aug 12;8(8):001145.v3. doi: 10.1099/acmi.0.001145.v3 (PMC13473831; doi:10.1099/acmi.0.001145.v3)
Supplement: Supplementary file 1 [file acmi-8-01145-s001.pdf]

Supplementary Table 1: Strains used in this study

Strain	Genotype[a]	Parent strain[b]	Plasmid used[c]	Source
SGB771	*W168 ΔbceS cm^r sacA::(PbceA-luxABCDE) spc^r*	-	-	[1]
SGB776	*W168 ΔbceS cm^r sacA::(PbceA-luxABCDE) spc^r lacA::(Pxyl-bceS^{WT}-His8) mls^r*	SGB771	pSD2E01	this work
SGB767	*W168 ΔbceS cm^r sacA::(PbceA-luxABCDE) spc^r lacA::(Pxyl-BceS^{T128A}-His8) mls^r*	SGB771	pAK2E140	this work
SGB768	*W168 ΔbceS cm^r sacA::(PbceA-luxABCDE) spc^r lacA::(Pxyl-BceS^{T128S}-His8) mls^r*	SGB771	pAK2E141	this work
SGB769	*W168 ΔbceS cm^r sacA::(PbceA-luxABCDE) spc^r lacA::(Pxyl-BceS^{T128Q}-His8) mls^r*	SGB771	pAK2E142	this work
SGB770	*W168 ΔbceS cm^r sacA::(PbceA-luxABCDE) spc^r lacA::(Pxyl-BceS^{T128N}-His8) mls^r*	SGB771	pAK2E143	this work

[a] *Bacillus subtilis* antibiotic resistance markers. *cm^r*, chloramphenicol resistance; *spc^r*, spectinomycin resistance; *mls^r*, macrolides, lincosamides, and streptogramins resistance.

[b] Parent strain indicates the recipient of the plasmid DNA.

[c] Plasmid DNA used in construction of the strain.

Supplementary Table 2: Plasmids used in this study

Plasmid	Genotype[a]	Parent plasmid[b]	Plasmid construction[c]	Source
pSD2E01	*bla^r lacA::(Pxyl-bceS^{WT}-His8) mls^r*	-	-	[2]
pAK2E140	*bla^r lacA::(Pxyl-bceS^{T128A}-His8) mls^r*	pSD2E01	SGB0514/SGB0515	this work
pAK2E141	*bla^r lacA::(Pxyl-bceS^{T128S}-His8) mls^r*	pSD2E01	SG0516/SG0517	this work
pAK2E142	*bla^r lacA::(Pxyl-bceS^{T128Q}-His8) mls^r*	pSD2E01	SG0518/SG0519	this work
pAK2E143	*bla^r lacA::(Pxyl-bceS^{T128N}-His8) mls^r*	pSD2E01	SG0520/SG0521	this work

[a] *E. coli* antibiotic resistance marker*;* *bla^r*, ampicillin. *B. subtilis* antibiotic resistance marker; *mls^r*, macrolides, lincosamides and streptogramins resistance.

[b] Parent plasmid indicates the template DNA used for mutagenesis.

[c] Primer pairs used in the mutagenesis with the parent plasmid as template DNA.

Supplementary Table 3: Oligonucleotides used in this study

Primer	Description	Sequence (5' → 3')[a]
SGB0514	*bceS*^{T128A}	CAAAgctCCATTAACAGCGATGCATTTAATC
SGB0515	*bceS*^{T128A}	AATGGagcTTTGACCTCATGGATCCATGCC
SGB0516	*bceS*^{T128S}	CAAAtctCCATTAACAGCGATGCATTTAATC
SGB0517	*bceS*^{T128S}	AATGGagaTTTGACCTCATGGATCCATGCC
SGB0518	*bceS*^{T128Q}	CAAAcaaCCATTAACAGCGATGCATTTAATC
SGB0519	*bceS*^{T128Q}	AATGGttgTTTGACCTCATGGATCCATGCC
SGB0520	*bceS*^{T128N}	CAAAaacCCATTAACAGCGATGCATTTAATC
SGB0521	*bceS*^{T128N}	AATGGgttTTTGACCTCATGGATCCATGCC

[a] Lower case indicates the point mutation.

Supplementary data references

1. Koh, A., et al., *Conformation control of the histidine kinase BceS of Bacillus subtilis by its cognate ABC-transporter facilitates need-based activation of antibiotic resistance.* Mol Microbiol, 2021. **115**(1): p. 157-174.

2. Dintner, S., et al., *A sensory complex consisting of an ATP-binding cassette transporter and a two-component regulatory system controls bacitracin resistance in Bacillus subtilis.* J Biol Chem, 2014. **289**(40): p. 27899-910.

BceS variants	Mean difference (Wild-type - variant)	Adjusted 95% CI	Adjusted P-value	Significance
Δ*bceS*	48320	36797 to 59842	0.0008	***
T128A	-10815	-20916 to -714.8	0.0385	*
T128S	23285	7633 to 38937	0.0099	**
T128N	20180	7129 to 33231	0.0067	**
T128Q	48396	36873 to 59918	0.0008	***

Fig. S1. Statistical analysis of wild-type BceS and BceS substitution mutants signalling assay. Welch's one-way ANOVA followed by Dunnett's T3 multiple comparisons test were employed. Mean differences were calculated as wild-type minus each mutant reporter activity; negative values indicate higher reporter activity compared to wild-type, while positive values indicate lower reporter activity compared to wild-type. Adjusted 95~% confidence intervals (CI) and multiplicity adjusted P-values are reported with ***, $p < 0.001$; **, $p < 0.01$; *, $p < 0.05$.

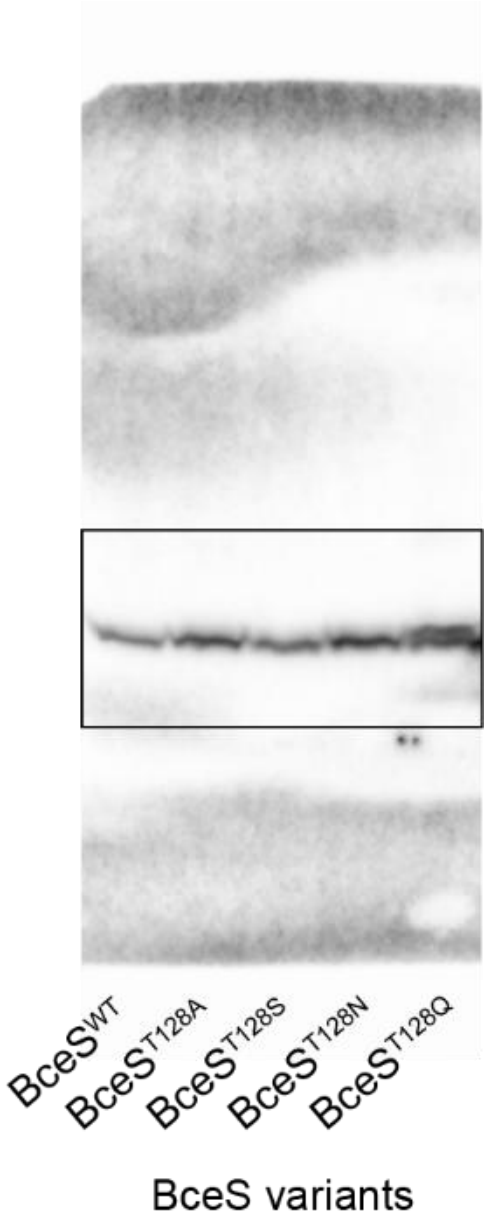

Fig. S2. Uncropped Western blot of His-tagged BceS substitution mutants detected by *anti-His* antibodies. Full length BceS was detected for all variants, with no detectable lower molecular weight degradation products. The black box indicates the region shown in Fig. 3c.
